# iMeta: Biology and medicine in informatics and MetaOmics era

**DOI:** 10.1002/imt2.70189

**Published:** 2026-11-13

**Authors:** Gousi Li, Qiao Yu, Zhihao Zhu, Xiaofang Yao, Yao Wang, Hao Luo, Defeng Bai, Yunyun Gao, Jiani Xun, Meiyin Zeng, Tianyuan Zhang, Tengfei Ma, Zi‐Ang Shen, Wei Han, Jiqiu Wu, Li‐Jing Bai, Feiying Zhu, Chun‐Sheng Hou, Lin Zhang, Cheng Gao, Renhong Yan, Yi Song, Guirong Wang, Shan‐shan Xu, Weihua Pan, Hao Zheng, Yue Sheng, Qingsong Ye, Jia‐Hui Liu, Chun Yin, Jialong Cao, Dong‐Rui Chen, Hanqing Chen, Hu Chen, Ji‐Kang Chen, Jiani Chen, Mingliang Chen, Ruibing Chen, Lei Cheng, Die Dai, Xi‐Jian Dai, Jianjun Deng, Xile Deng, Na Ding, Xia Ding, Zequan Ding, Xuan Dong, Yong‐Zhen Dong, Jiajia Duan, Yan Gao, Yi‐Zhou Gao, Ya‐Ting Ge, Wenping Gong, Harvest F. Gu, Rui Guo, Xiang Guo, Ying‐ying Han, Jiu‐Xing He, Xiaoqing He, Haiming Hu, Jianhua Huang, Roujie Huang, Yuansheng Huang, Di Jiang, Mengxi Jiang, Tao Jiang, Juan Lai, Bo Li, Huibo Li, Jian‐Feng Li, Jian Li, Ruichao Li, Shuai Li, Wei Li, Xiaojing Li, Xuemeng Li, Yin Li, Zhan‐zhan Li, Jie Liao, Xing‐Hua Liao, Biao Liu, Bingqi Liu, Sheng Liu, Tianyang Liu, Wen‐Hui Liu, Yi Liu, Zhipeng Liu, Hong‐Ye Lu, Hui Lu, Xiao Luo, Haiquan Lu, Wei Ma, Hongfeng Mao, Wenjun Mao, Ming Nie, Huili Pang, Yan Pang, Ziqin Pang, Hui Qu, Cheng Shen, Chuankui Song, Xiaoming Song, Qi Su, Hao Sun, Xinyi Sun, Yan Sun, Xiaoqiang Tang, Bowen Wang, Dingjie Wang, Dong Wang, Gui‐Wei Wang, Hanfei Wang, Nanqi Wang, Shixiang Wang, Tong Wang, Xi Wang, Xinyuan Wang, Yufeng Wang, Yongjun Wei, Shu Wen, Peng Wu, Shengbo Wu, Yuan Xiang, Haiqing Xiao, Jian Xiao, Weihua Xiao, Jun Yan, Bingsheng Yang, Haixia Yang, Xin Yang, Ze‐Wei Yang, Shi‐Jie Yao, Hengchuang Yin, Yang yan Yin, Li Yu, Xinxin Yu, Can Zhang, Chi Zhang, Deyong Zhang, Jian Zhang, Jinping Zhang, Jingsong Zhang, Peng Zhang, Yu Zhang, Pingsen Zhao, Tianyi Zhao, Xue‐Ming Zhao, Yuchao Zhao, Yucheng Zhao, Ruihao Zhou, Tao Zhou, Xin Zhou, Yongmei Zhou, Siyuan Zhu, Xiusheng Zhu, Yilin Xu, Xiong‐Feng Ma, Lianyang Bai, Zhenmin Bao, Shuang‐Jiang Liu, Kui Li, Suyin Feng, Ziheng Wang, Peng Luo, Ren‐You Gan, Gang Chen, Yulong Yin, Tong Chen, Yong‐Xin Liu

**Affiliations:** ^1^ State Key Laboratory of Tropical Crop Breeding, Genome Analysis Laboratory of the Ministry of Agriculture and Rural Affairs, Agricultural Genomics Institute at Shenzhen, Chinese Academy of Agricultural Sciences Shenzhen Guangdong China; ^2^ School of Ecology and Nature Conservation Beijing Forestry University Haidian District Beijing China; ^3^ Benagen Institute Wuhan Hubei China; ^4^ State Key Laboratory of Herbage Improvement and Grassland Agro‐ecosystems, Centre for Grassland Microbiome, College of Pastoral Agriculture Science and Technology Lanzhou University Lanzhou Gansu China; ^5^ College of Food Science and Engineering Central South University of Forestry and Technology Changsha Hunan China; ^6^ CAS Key Laboratory of Pathogen Microbiology and Immunology, Institute of Microbiology, Chinese Academy of Sciences (CAS) Beijing China; ^7^ Agricultural Genomics Institute at Shenzhen, Chinese Academy of Agricultural Sciences Shenzhen Guangdong China; ^8^ Hunan Academy of Agricultural Sciences Changsha China; ^9^ Institute of Bast Fiber Crops, Chinese Academy of Agricultural Sciences Changsha Hunan China; ^10^ Hubei Shizhen Laboratory, School of Basic Medical Sciences Hubei University of Chinese Medicine Wuhan China; ^11^ State Key Laboratory of Microbial Diversity and Innovative Utilization, Institute of Microbiology, Chinese Academy of Sciences Beijing China; ^12^ Southern University of Science and Technology Shenzhen Guangdong China; ^13^ Shenzhen Key Laboratory of Plant Genetic Engineering and Molecular Design, Institute of Plant and Food Science, Department of Biology, School of Life Sciences Southern University of Science and Technology Shenzhen China; ^14^ Institute of Agro‐Products Processing Research Anhui Academy of Agricultural Sciences Hefei China; ^15^ College of Food Science and Nutritional Engineering China Agricultural University Beijing China; ^16^ The Second Xiangya Hospital of Central South University Changsha Hunan China; ^17^ Center of Regenerative Medicine, Department of Stomatology Renmin Hospital of Wuhan University Wuhan China; ^18^ State Key Laboratory of Genetics and Development of Complex Phenotypes, Institute of Plant Biology, School of Life Sciences Fudan University Shanghai China; ^19^ Equus Research Center Inner Mongolia Agricultural University Hohhot China; ^20^ State Key Laboratory for Animal Disease Control and Prevention South China Agricultural University Guangzhou Guangdong China; ^21^ Beijing Key Laboratory of Environment and Aging, Department of Nutrition and Food Hygiene, School of Public Health Capital Medical University Beijing China; ^22^ Zhejiang Key Laboratory of Biology and Ecological Regulation of Crop Pathogens and Insects, Institute of Insect Sciences, College of Agriculture and Biotechnology Zhejiang University Hangzhou Zhejiang China; ^23^ Department of Clinical Nutrition, Southwest Hospital Army Medical University (Third Military Medical University) Chongqing China; ^24^ Department of Pharmaceutical Botany, School of Pharmacy Naval Medical University Shanghai China; ^25^ State Key Laboratory for Vegetation Structure, Function and Construction, MOE Key Laboratory of Biosystems Homeostasis & Protection, College of Life Sciences Zhejiang University Hangzhou China; ^26^ Chinese Medicine Guangdong Laboratory Guangzhou University of Chinese Medicine Hengqin Guangdong China; ^27^ Department of Medical Imaging The First Affiliated Hospital of Gannan Medical University Ganzhou Jiangxi China; ^28^ State Key Laboratory of Vegetable Biobreeding, Institute of Vegetables and Flowers, Chinese Academy of Agricultural Sciences Beijing China; ^29^ Yuelushan Laboratory, Hunan Academy of Agricultural Sciences Changsha China; ^30^ Harbin Medical University Harbin China; ^31^ Nanchang University Nanchang China; ^32^ Department of Pediatric Surgery Children's Hospital of Nanjing Medical University Nanjing Jiangsu China; ^33^ Yellow Sea Fisheries Research Institute, Chinese Academy of Fishery Sciences Qingdao China; ^34^ Academy of Food Interdisciplinary Science Dalian Polytechnic University Dalian China; ^35^ Luoyang Key Laboratory of Zebrafish Toxicology Research, Luoyang Key Laboratory of Transplantation and Immunological Studies for Hematological Diseases, Department of Clinical Laboratory, the First Affiliated Hospital College of Clinical Medicine of Henan University of Science and Technology Luoyang Henan China; ^36^ Harbin Institute of Technology Harbin China; ^37^ Department of Environmental Hygiene, School of Public Health Harbin Medical University Harbin China; ^38^ State Key Laboratory of Bioactive Substance and Function of Natural Medicines, Institute of Materia Medica, Chinese Academy of Medical Sciences and Peking Union Medical College Beijing China; ^39^ Senior Department of Tuberculosis Chinese PLA General Hospital Beijing China; ^40^ Laboratory of Molecular Medicine, School of Basic Medicine and Clinical Pharmacy China Pharmaceutical University Nanjing Jiangsu China; ^41^ State Key Laboratory for Quality and Safety of Agro‐Products, Institute of Environment, Resource, Soil and Fertilizer, Zhejiang Academy of Agricultural Sciences Hangzhou China; ^42^ Department of Pathogen Biology, School of Basic Medical Sciences Henan University Kaifeng China; ^43^ College of Food Science and Engineering Shandong Agricultural University Tai'an Shandong China; ^44^ Xianghu Laboratory Hangzhou China; ^45^ State Key Laboratory of Efficient Production of Forest Resources, College of Biological Sciences and Technology Beijing Forestry University Beijing China; ^46^ School of Laboratory Medicine Hubei University of Chinese Medicine Wuhan Hubei China; ^47^ National Clinical Research Center for Women's Health and Obstetric and Gynecologic Diseases, Department of Obstetrics and Gynecology State Key Laboratory of Common Mechanism Research of Major Diseases, Peking Union Medical College Hospital, Chinese Academy of Medical Sciences and Peking Union Medical College Beijing China; ^48^ Henan University of Technology Zhengzhou Henan China; ^49^ Department of Pharmacology, School of Basic Medical Sciences Capital Medical University Fengtai District Beijing China; ^50^ Faculty of Computing, Harbin Institute of Technology Harbin China; ^51^ GeneMind Biosciences Company Limited Shenzhen Guangdong China; ^52^ School of Medicine and Health, Harbin Institute of Technology Harbin Heilongjiang China; ^53^ Department of Pharmacy Peking University Third Hospital Beijing China; ^54^ Shanghai Institute of Hematology, State Key Laboratory of Medical Genomics, National Research Center for Translational Medicine at Shanghai, Ruijin Hospital Shanghai Jiao Tong University School of Medicine Shanghai China; ^55^ Jiangxi Province Key Laboratory of Pharmacology of Traditional Chinese Medicine, State Key Laboratory of New Targets Discovery and Drug Development for Major Diseases, School of Pharmacy Gannan Medical University Ganzhou Jiangxi China; ^56^ Jiangsu Co‐Innovation Center for Prevention and Control of Important Animal Infectious Diseases and Zoonoses, College of Veterinary Medicine Yangzhou University Yangzhou China; ^57^ Laboratory of Brewing Microbiology and Applied Enzymology, Key Laboratory of Industrial Biotechnology of Ministry of Education, School of Biotechnology Jiangnan University China; ^58^ Shenzhen Bay Laboratory, Pingshan Translational Medicine Center Shenzhen China; ^59^ Zhanjiang Key Laboratory of Human Microecology and Clinical Translation Research, the Marine Biomedical Research Institute, School of Basic Medicine Guangdong Medical University Zhanjiang Guangdong China; ^60^ The International Science and Technology Cooperation Base (Genetic Engineering) of Chinese Ministry of Science and Technology, the Key Laboratory of Molecular Biophysics of Chinese Ministry of Education, College of Life Science and Technology Huazhong University of Science and Technology Wuhan China; ^61^ Academy of Chinese Medicine Sciences Henan University of Chinese Medicine China; ^62^ State Key Laboratory of Chinese Medicine Modernization, Innovation Center of Yangtze River Delta Zhejiang University Jiaxing Zhejiang China; ^63^ Institute of Biology and Medicine, College of Life and Health Science Wuhan University of Science and Technology Wuhan China; ^64^ Hunan Institute of Microbiology Hunan Academy of Agricultural Sciences Changsha Hunan China; ^65^ Jiangxi Provincial Key Laboratory of Pest and Disease Control of Featured Horticultural Plants Gannan Normal University Ganzhou Jiangxi China; ^66^ The Seventh Affiliated Hospital of Sun Yat‐sen University Shenzhen Guangdong China; ^67^ Department of Microbiology, College of Life Science Shandong Agricultural University Tai'an Shandong China; ^68^ Collaborative Innovation Center for Zhejiang Marine High‐efficiency and Healthy Aquaculture, School of Marine Sciences Ningbo University Ningbo Zhejiang China; ^69^ State Key Laboratory of Herbage Improvement and Grassland Agro‐Ecosystems, College of Pastoral Agriculture Science and Technology Lanzhou University Lanzhou Gansu China; ^70^ Stomatology Hospital, School of Stomatology Zhejiang University School of Medicine; Clinical Research Center for Oral Diseases of Zhejiang Province; Key Laboratory of Oral Biomedical Research of Zhejiang Province; Cancer Center of Zhejiang University Hangzhou Zhejiang China; ^71^ Department of Pharmacy, Shanghai Tenth People's Hospital, School of Medicine Tongji University Shanghai China; ^72^ Hunan Research Center of the Basic Discipline for Cell Signaling, College of Biology Hunan University Changsha Hunan China; ^73^ Advanced Medical Research Institute, Cheeloo College of Medicine Shandong University Jinan Shandong China; ^74^ State Key Laboratory of North China Crop Improvement and Regulation, Key Laboratory of Vegetable Germplasm Innovation and Utilization of Hebei, College of Horticulture Hebei Agricultural University Baoding Hebei China; ^75^ State Key Laboratory of Metabolic Dysregulation & Prevention and Treatment of Esophageal Cancer, Shanghai Key Laboratory of Diabetes Mellitus, Department of Endocrinology and Metabolism, Shanghai Diabetes Institute, Shanghai Clinical Center for Diabetes Shanghai Sixth People's Hospital Affiliated to Shanghai Jiao Tong University School of Medicine Shanghai China; ^76^ Department of Thoracic Surgery, the Affiliated Wuxi People's Hospital of Nanjing Medical University, Wuxi People's Hospital, Wuxi Medical Center Nanjing Medical University Wuxi China; ^77^ National Observations and Research Station for Wetland Ecosystems of the Yangtze Estuary, School of Life Sciences Fudan University Shanghai China; ^78^ State Key Laboratory of Cotton Bio‐breeding and Integrated Utilization, School of Agriculture and Biomanufacturing Zhengzhou University Zhengzhou China; ^79^ Institute of Grassland Research, Chinese Academy of Agricultural Sciences Hohhot Inner Mongolia China; ^80^ State Key Laboratory of Tea Plant Germplasm Innovation and Resource Utilization Anhui Agricultural University Hefei China; ^81^ School of Life Sciences North China University of Science and Technology Tangshan Hebei China; ^82^ Department of Medicine and Therapeutics Chinese University of Hong Kong China; ^83^ Wolfson Institute for Biomedical Research, UCL Division of Medicine, University College London, Gower St London UK; ^84^ Mingqian Institute of Microbiology Suzhou Jiangsu China; ^85^ Chinese National Microbiology Data Center (NMDC) Beijing China; ^86^ Key Laboratory of Birth Defects and Related Diseases of Women and Children of MOE West China Second University Hospital, Sichuan University Chengdu China; ^87^ Guojiu Research Institute, School of Food and Health Beijing Technology and Business University Beijing China; ^88^ Institute for Math & AI Wuhan University Wuhan China; ^89^ College of Veterinary Medicine Shandong Agricultural University Tai'an Shandong China; ^90^ National Engineering Research Center for Efficient Utilization of Soil and Fertilizer Resources, College of Resources and Environment Shandong Agricultural University Taian Shandong China; ^91^ College of Animal Science and Technology Yangtze University Jingzhou Hubei China; ^92^ National Citrus Engineering Research Center, Chongqing Key Laboratory of Citrus, Citrus Research Institute Southwest University Chongqing China; ^93^ Department of Biomedical Informatics, School of Life Sciences Central South University Changsha Hunan China; ^94^ Hunan Kaiyou Biotechnology Co., Ltd. Changsha Hunan China; ^95^ State Key Laboratory of Reproductive Medicine and Offspring Health Nanjing Medical University Nanjing China; ^96^ College of Animal Science Inner Mongolia Agricultural University Hohhot Inner Mongolia China; ^97^ School of Pharmaceutical Sciences, Key Laboratory of Advanced Drug Preparation Technologies, Ministry of Education Zhengzhou University Zhengzhou China; ^98^ Wensyn Life Science Co., Ltd. Wuxi China; ^99^ Department of Nephrology The First Affiliated Hospital of Zhengzhou University Zhengzhou Henan China; ^100^ Zhejiang Institute of Tianjin University (Shaoxing) Shaoxing China; ^101^ Department of Medical Laboratory, The Central Hospital of Wuhan, Tongji Medical College Huazhong University of Science and Technology Wuhan Hubei China; ^102^ State Key Laboratory of Vaccines for Infectious Diseases, National Institute of Diagnostics and Vaccine Development in Infectious Diseases, Xiang An Biomedicine Laboratory, School of Life Sciences & School of Public Health Xiamen University Xiamen Fujian China; ^103^ College of Agriculture Hunan Agricultural University Changsha China; ^104^ Beilun People's Hospital Ningbo Zhejiang China; ^105^ College of Agronomy and Biotechnology China Agricultural University Beijing China; ^106^ Department of Joint and Orthopedics, Zhujiang Hospital Southern Medical University Guangzhou Guangdong China; ^107^ College of Food Science and Nutritional Engineering China Agricultural University China; ^108^ State Key Laboratory for Quality and Safety of Agro‐Products Ningbo University Ningbo China; ^109^ School of Urban Construction & Safety Engineering, Shanghai Institute of Technology Shanghai China; ^110^ Xinjiang Technical Institute of Physics and Chemistry, Chinese Academy of Sciences Urumqi Xinjiang China; ^111^ College of Animal Science and Technology Guangxi University Nanning Guangxi China; ^112^ School of Life Sciences, State Key Laboratory for Conservation and Utilization of Bio‐Resource in Yunnan Yunnan University Kunming China; ^113^ Department of Orthopaedics The Second Qilu Hospital of Shandong University, Shandong University Centre for Orthopaedics, Cheeloo College of Medicine, Shandong University Jinan China; ^114^ State Key Laboratory of Hybrid Rice, Institute of Plant Protection, Hunan Academy of Agricultural Sciences Changsha China; ^115^ Department of Gastroenterology the First Affiliated Hospital (Southwest Hospital) of Third Military Medical University (Army Medical University) Chongqing China; ^116^ Pediatric Department, Sixth People's Hospital Jiao Tong University Shanghai China; ^117^ Faculty of Military Health Services Naval Medical University Shanghai China; ^118^ Department of Environment, College of Environment and Resources Xiangtan University Xiangtan China; ^119^ Guangdong Provincial Key Laboratory of Marine Biotechnology, Department of Biology, College of Science Shantou University Shantou Guangdong China; ^120^ Yuebei People's Hospital Affiliated to Shantou University Medical College Shaoguan Guangdong China; ^121^ Institute of Animal Science, Chinese Academy of Agricultural Sciences Beijing China; ^122^ College of Animal Science and Technology Beijing University of Agriculture Beijing China; ^123^ School of Pharmacy Nanjing University of Chinese Medicine Nanjing China; ^124^ Department of Anesthesiology, The Research Units of West China (2018RU012)‐the Anesthesiology & Intelligence Research Collaboration Group (AIR‐CG), West China Hospital Sichuan University Chengdu Sichuan China; ^125^ Faculty of Life and Health Sciences Shenzhen University of Advanced Technology Shenzhen China; ^126^ Department of Genetics and Bioinformatics, School of Life Sciences Anhui Medical University Hefei Anhui China; ^127^ Hunan Academy of Agricultural Sciences Changsha Hunan China; ^128^ MOE Key Laboratory of Marine Genetics and Breeding, Qingdao Institute of Blue Seed Industry Ocean University of China Qingdao China; ^129^ State Key Laboratory of Microbial Technology Shandong University Jinan Shandong China; ^130^ Cardio‐Cerebral Vascular Disease Prevention and Treatment Innovation Center, Donghai County People's Hospital (Affiliated Kangda College of Nanjing Medical University) Lianyungang Jiangsu China; ^131^ Department of Rehabilitation Sciences, Faculty of Health and Social Sciences The Hong Kong Polytechnic University Hung Hom Hong Kong SAR China; ^132^ Department of Oncology, Zhujiang Hospital Southern Medical University Guangzhou Guangdong China; ^133^ Department of Food Science and Nutrition The Hong Kong Polytechnic University Kowloon Hong Kong SAR China; ^134^ Hubei University of Chinese Medicine, Hubei Shizhen Laboratory, Department of Geriatrics Affiliated Hospital of Hubei University of Chinese Medicine Wuhan China; ^135^ Hunan Provincial Key Laboratory of Animal Nutritional Physiology and Metabolic Process, National Engineering Laboratory for Pollution Control and Waste Utilization in Livestock and Poultry Production, Institute of Subtropical Agriculture Chinese Academy of Sciences Changsha Hunan China; ^136^ State Key Laboratory for Quality Ensurance and Sustainable Use of Dao‐di Herbs, National Resource Center for Chinese Materia Medica, Institute of Chinese Materia Medica, China Academy of Chinese Medical Sciences Beijing China

## Abstract

This study presents a decade‐long bibliometric map of informatics and metaomics research in biology and medicine, spanning life and agricultural sciences, basic and translational medicine, methodology innovation, and interdisciplinary frontiers. Aligning these findings with insights from the iMeta Conference 2026, which fostered talent cultivation and academic exchange among global researchers, we further highlight how the iMeta journal community captures the challenges and opportunities in this rapidly evolving era.

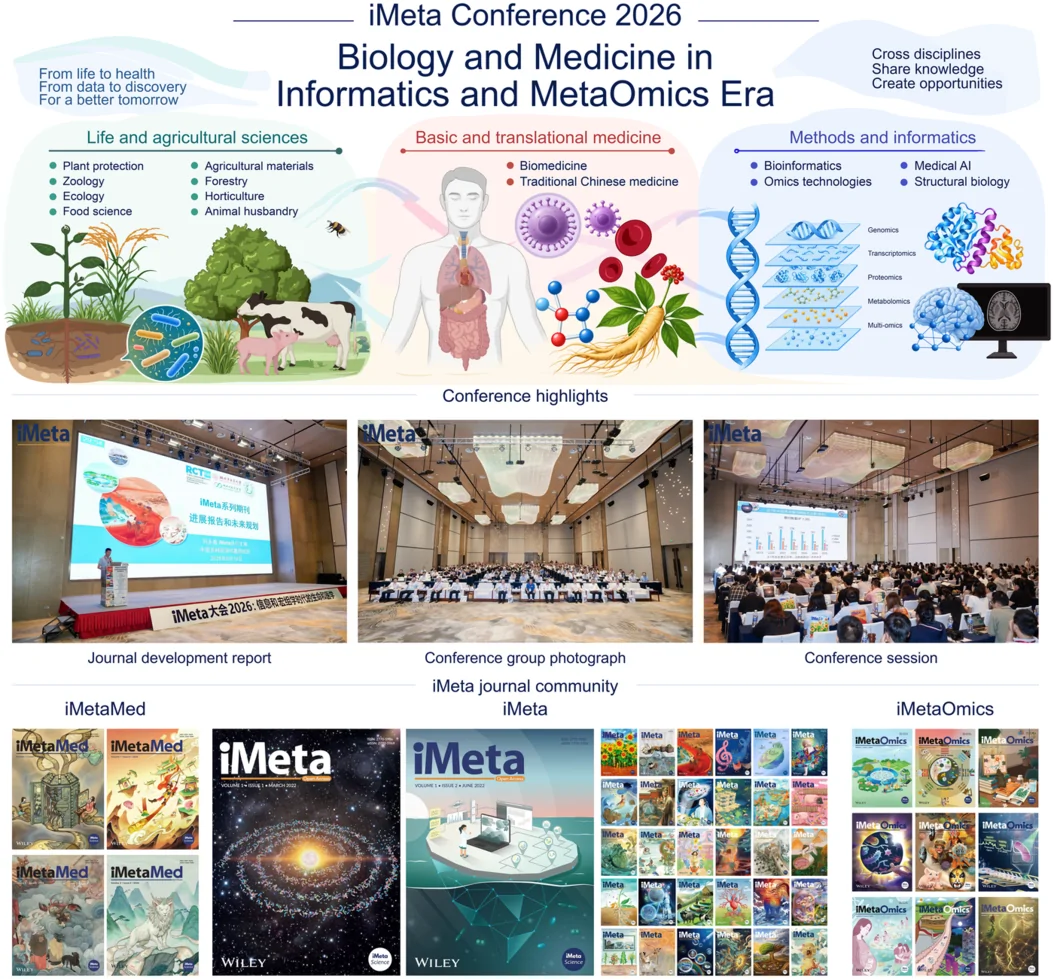

Biological and medical research is undergoing a fundamental paradigm shift from “hypothesis‐driven” to “data‐driven” science, with the rapid growth of high‐throughput technologies and computational capacity. Unlike traditional approaches that focus on single molecules, MetaOmics, which includes single and integrated genomics, transcriptomics, proteomics, metabolomics, etc., offers a holistic and integrated view of biological systems by massive‐data collection, and has been widely applied in various biological and medical research fields in the past two decades [1, 2]. Informatics, spanning bioinformatics, health informatics, and artificial intelligence (AI), provides computational tools to decipher complex datasets and networks, bridging the gap between raw data and scientific insights [3]. Since the launch of the Human Genome Project, Informatics and MetaOmics have emerged as two strong forces reshaping life sciences and medical sciences.

To capture the boost of studies in the Informatics and MetaOmics era, iMeta and its series of journals—iMetaOmics, iMetaMed, and the upcoming ones— were established as a timely platform fostering interdisciplinary development in biology, medicine, and informatics [4, 5]. Besides, the annual iMeta Conference has consistently served as a key hub for disseminating world‐class technological advancements in biology and medicine [6, 7]. Despite the rapid advancement in these fields, there is a pressing need to understand the developmental trends of the past decade that navigate the future landscape. Here, we perform a decade of bibliometric analysis to quantify the global research landscape and provide a comprehensive overview of the field (Figure S1). By aligning these findings with insights from the iMeta Conference 2026, we aim to offer a future perspective on the challenges and opportunities in this rapidly evolving era.

## OVERVIEW OF INFORMATICS AND METAOMICS IN GLOBAL BIOLOGICAL AND MEDICAL RESEARCH

To quantify the adoption of informatics and metaomics in global biological and medical studies in the last decade, we first searched all the Article and Review records published from 2016 to 2025 in biological and medical disciplines across human, animal, plant, and environmental ecosystems (Tables S1 and S2). The adoption of informatics and metaomics was identified via keyword analysis (Tables S3 and S4). While the total publication records increased gradually from 2016 to 2021 and reached a plateau, the proportion of informatics and metaomics applications continuously increased from 8.8% to 22.1% over the decade (Figure 1A). This trend indicates that informatics and metaomics are undergoing a period of rapid expansion.

**FIGURE 1:**
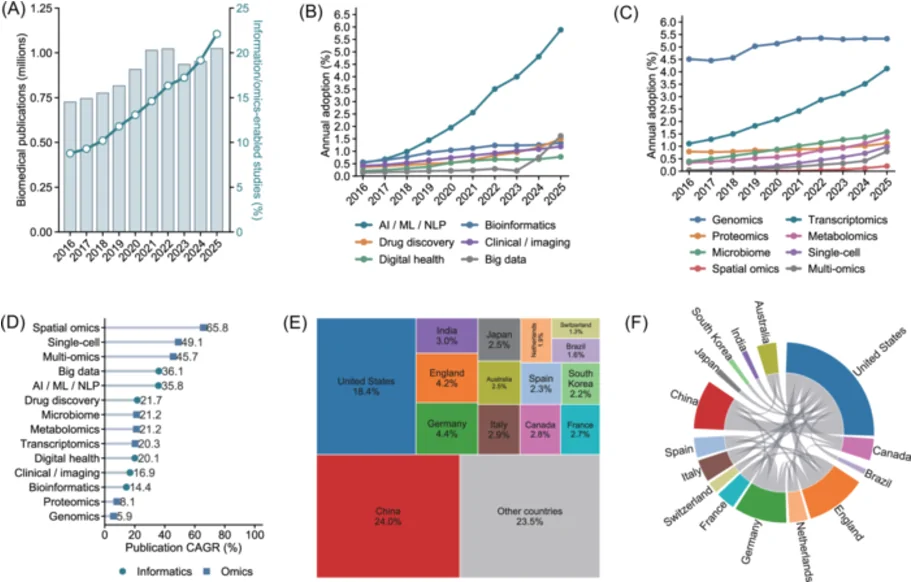
Overview of Informatics and MetaOmics in global biological and medical research. (A) Trends in annual publications in biology and medicine (left axis) and the adoption rate of informatics and metaomics (right axis) from 2016 to 2025. (B) Annual adoption rates of six representative categories in informatics. (C) Annual adoption rates of eight representative categories in metaomics. (D) Ranked compound annual growth rates (CAGRs) of the representative categories in informatics and metaomics. (E) Global distribution of informatics and metaomics publications from 2016 to 2025. (F) Global collaboration network for informatics and omics publications from 2016 to 2025.

To clarify the development of specific areas of informatics and metaomics, we further focus on six categories of informatics, including AI, bioinformatics, computational drug discovery, clinical and imaging informatics, digital health, and big data; eight categories of metaomics, which include genomics, transcriptomics, proteomics, metabolomics, metagenomics/microbiome, single‐cell omics, spatial omics, and multi‐omics. Among the informatics categories, all the six categories showed close adoption rates in 2016 (0.14%–0.56%), whereas AI‐related publications increased dramatically from 0.53% in 2016 to 5.89% in 2025, becoming the dominant category (Figure 1B). In addition, publications related to big data experienced a substantial increase after 2023, while the other categories maintained a slow growth over the decade (Figure 1B). For the metaomics categories, although genomics research entered a plateau after 2021, it still occupies a dominant position among all omics studies (Figure 1C). By contrast, metagenomics/microbiome, metabolomics, and proteomics maintained a steady growth over the decade, and the publication of three emerging fields—single‐cell omics, spatial omics, and multi‐omics—also showed a significant growth since 2020 (Figure 1C). Notably, transcriptomics increased substantially from 1.11% to 4.13% during the last decade (Figure 1C), probably due to its deep integration with new technologies such as single‐cell omics and spatial omics [8], and the decrease in sequencing costs [9]. The annual growth rates further showed that most of the emerging fields, such as spatial omics, single‐cell omics, multi‐omics, big data, and AI, had the highest growth rates (65.8%, 49.1%, 45.7%, 36.1%, and 35.8%, respectively), compared with the lowest growth rate of the traditional fields (8.1% of proteomics and 5.9% of genomics) (Figure 1D).

The global distribution of informatics‐ and omics‐enabled research is concentrated but internationally connected (Figure 1E,F). China and the United States accounted for 24.0% and 18.4% of total country‐participation records over 2016–2025, followed by Germany (4.4%), England (4.2%), India (3.0%), Italy (2.9%), Canada (2.8%), France (2.7%), Australia (2.5%), Japan (2.5%), Spain (2.3%), and South Korea (2.2%). China—the United States collaboration is the strongest bilateral link among all the countries, and the United States also formed prominent links with England, Germany, and Canada.

## HIGH‐IMPACT STUDIES CONVERGED ON FIVE INTERCONNECTED THEMES

We next obtained 1000 high‐impact informatics‐ and omics‐enabled studies published during 2016–2025 based on citation counts for research topic hotspot analysis. The co‐occurrence network of high‐frequency terms identified five themes: microbiome and host health, cancer and clinical translation, AI and clinical data analysis, bioinformatics resources and molecular prediction, and COVID‐19 and population health (Figure 2A). Among the 1000 high‐impact studies, 968 records were classified into at least one theme, and 32 remained unclassified. Besides, 663 publications are mapped to two or more themes, indicating significant overlap in these research themes. To further demonstrate the interconnection among the five themes, we calculated the coverage of studies for each individual theme, as well as the overlap between any two themes (Figure 2B). The strongest pairwise overlap occurred between microbiome & host health and cancer & clinical translation (245 shared studies), cancer & clinical translation and AI & clinical data (226 shared studies), and AI & clinical data and bioinformatics resources & molecular prediction (190 shared studies). These results suggest that current research hotspots are driven by the integration of biological questions, informatics technologies, and translational applications.

**FIGURE 2:**
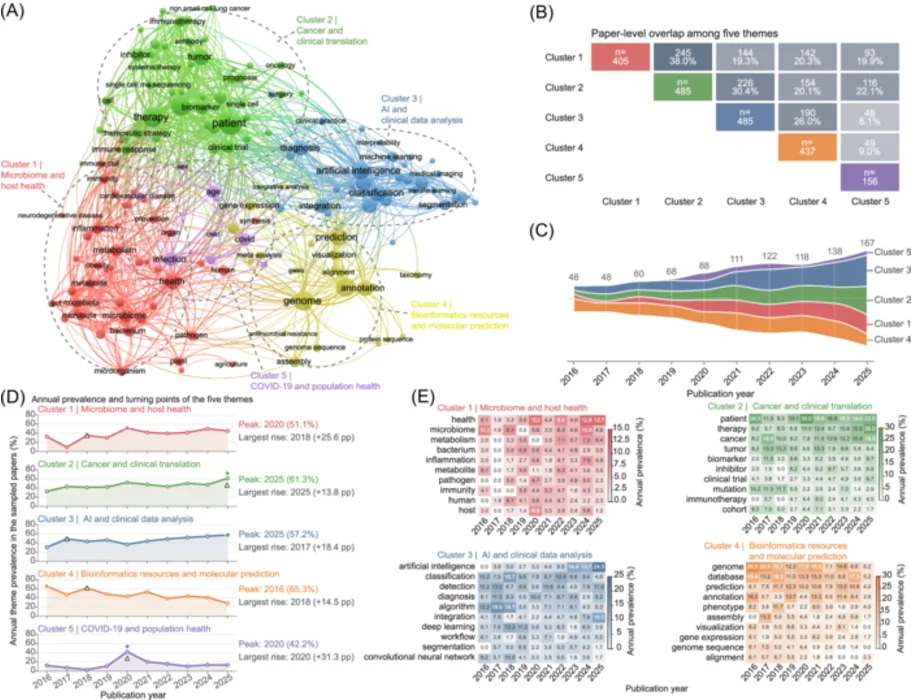
High‐impact studies converged on five interconnected themes. (A) Co‐occurrence network of high‐frequency terms from 1000 high‐impact informatics‐ and omics‐enabled studies published during 2016–2025. Nodes are sized by occurrence and colored by VOSviewer cluster membership (the five themes); connecting lines indicate co‐occurrence in the same article. (B) Paper‐level overlap matrix among the five themes. Diagonal cells indicate the number of articles assigned to each theme: *n* = 405 (Cluster 1, microbiome and host health), 485 (Cluster 2, cancer and clinical translation), 485 (Cluster 3, AI and clinical data analysis), 437 (Cluster 4, bioinformatics resources and molecular prediction), and 156 (Cluster 5, COVID‐19 and population health). Off‐diagonal cells indicate the number of shared articles (above) and the Jaccard similarity (below, intersection/union). (C) A streamgraph showing annual fractionally counted publications by theme from 2016 to 2025. A publication assigned to k themes contributes 1/k to each theme. Numbers above the stream indicate the annual number of publications assigned to at least one theme. (D) Annual percentage of selected publications assigned to each theme from 2016 to 2025, using all selected publications in the corresponding year as the denominator. The star marks the peak year; the triangle marks the year of the largest year‐on‐year increase in percentage points. (E) Annual prevalence (%) of the top 10 terms in Clusters 1–4 from 2016 to 2025, using all selected publications in the corresponding year as the denominator. Cluster 5 (COVID‐19 and population health) was excluded due to its small size (<10 terms in total).

Overall, the annual number of high‐impact publications assigned to these five themes increased from 48 in 2016 to 167 in 2025 (Figure 2C). However, the five themes exhibited distinct annual publication trends over the decade (Figure 2C,D). The peak of studies in COVID‐19 & population health was observed in 2020, consistent with the COVID‐19 outbreak, which also corroborates the credibility of our dataset. Although bioinformatics resources & molecular prediction contributed 65.3% of the total publications in 2016, the publication ratio for this theme gradually declined to 28.3% in 2025. In contrast, the publication ratio for the other three themes steadily increased over the decade, especially cancer & clinical translation and AI & clinical data analysis, which reached the highest proportions in 2025. These findings show that research interest in COVID‐19 and bioinformatics resources is cooling down, while interest in cancer, clinical translation and AI is on the rise.

We further calculated the annual frequency of the top 10 terms from the major themes to provide a keyword‐level perspective on research hotspots (Figure 2E). Terms regarding human diseases and health (e.g., health, patient, therapy, cancer, and tumor) consistently maintained high prominence, particularly terms associated with clinical translation, which showed an ascending trend. In addition, AI showed the largest percentage‐point increase in annual prevalence among the displayed terms over the past decade, rising from zero mention in 2016 high‐impact studies to one of the most frequent terms in 2025. Conversely, bioinformatics resources terms such as genome, database, and annotation exhibited a significant decrease from 2016 to 2025. Taken together, these patterns suggest a shift within the high‐impact studies from foundational sequencing and resource development towards AI‐enabled integration and clinically oriented applications, with a brief pandemic‐related expansion around 2020.

## IMETA CONFERENCE 2026

Since the founding of iMeta, four iMeta Conference series have been held in Qingdao, Beijing, Shenzhen, and Wuhan from 2022 to 2025 [6, 7]. The conference is dedicated to promoting cutting‐edge academic exchange, advancing disciplinary construction, as well as guiding the development of scientific research hubs. Continuing this mission, the iMeta Conference 2026 has just successfully concluded in Changsha (Figure 3), expanding its scope under the theme: “Biology and Medicine in Informatics and MetaOmics Era.”

**FIGURE 3:**
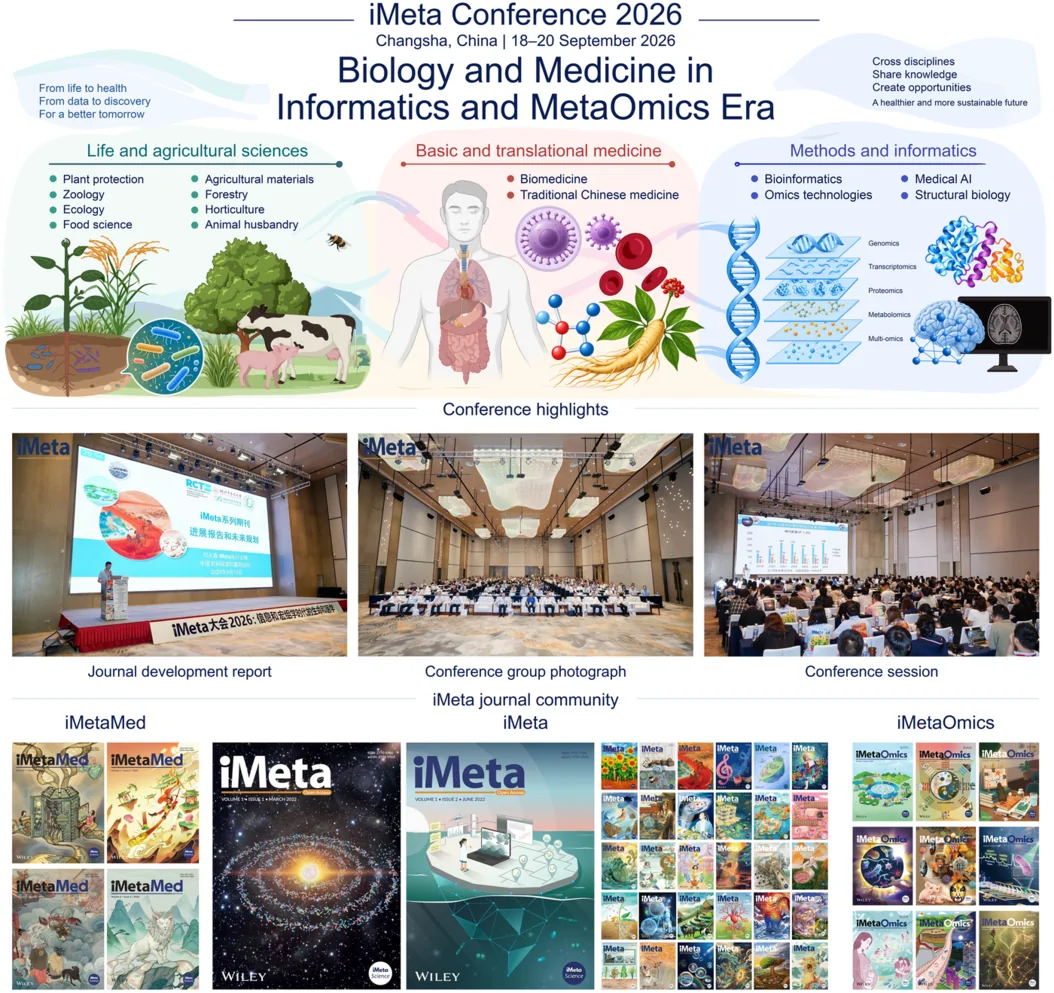
iMeta Conference 2026. Top: The conference encompassed life and agricultural sciences, basic and translational medicine, methodologies and informatics, as well as cutting‐edge interdisciplinary frontiers across these fields. Middle: Representative photographs from the conference, highlighting talent cultivation and academic exchange throughout the event. Bottom: The iMeta journal community serves as a dissemination platform for research findings in these fields.

To reflect this broad scope of iMeta, the conference included 18 specialized symposia within four key topics: the broad life and agricultural sciences, spanning plant protection, zoology, ecology, food science, agricultural materials, forestry, horticulture, and animal husbandry; the foundational and translational medicine, featuring biomedicine and traditional Chinese medicine; the methodological and informatical frontiers, including bioinformatics, omics technologies, medical AI, and biophysics; talent development and community building, encompassing the young scientists forum, the graduate students forum, the talent growth forum, and the editorial board meeting.

The event attracted over 600 participants across China. Among them, over 100 distinguished scientists who have made outstanding contributions to the interdisciplinary frontiers of life and medical sciences, gathered to share their latest findings in life and agricultural sciences, basic and translational medicine, methodologies and informatics [10]. In particular, the keynote and invited presentations highlighted the extensive integration of informatics and metaomics into livestock breeding, aquaculture, agricultural ecology, and medicine [11, 12, 13, 14, 15, 16, 17]. This gathering in Changsha served not only as a strategic milestone in strengthening the iMeta community but also as a compass for the evolution of biology and medicine.

## FUTURE PERSPECTIVES

The emergence of new technologies has consistently catalyzed rapid advances in science. Genome sequencing has led the life and medical sciences into the omics era since 2001, when the draft human genome sequence was published. Although genomics‐related studies have reached a plateau over the past 5 years, their publication output still far exceeds that of any other omics (Figure 1C), indicating the fundamental role of genomics in omics‐based research. Meanwhile, emerging technologies such as single‐cell sequencing, spatial multi‐omics, and AI are exhibiting dramatic growth and driving new trends in the field: traditional bulk omics is evolving toward higher‐resolution and integrated omics, while classical bioinformatics is advancing toward AI‐driven informatics.

The five interconnected themes shown in Figure 2A consistently focus on the health and development of humans, animals, plants, and the microbiome, indicating that “One Health” has become a major theme in current biological and medical research [18, 19]. With the explosive accumulation of omics and informatics resources, the research hotspot is undergoing a shift from large‐scale data collection toward in‐depth data integration for translational applications in production and clinical practice, as illustrated in Figure 2D. In the future, the central challenge of omics and informatics will no longer be the generation and interpretation of big data, but rather the transformation of such data into novel biological insights, driving progress in precision medicine, sustainable agriculture, and beyond, to achieve One Health.

Although China has established a leading position in publication output within informatics and metaomics, the proportion and diversity of its international collaborations still need to improve (Figure 1E,F). Recognizing this gap, iMeta Science is committed to bridging Chinese researchers with the global scientific community by launching a series of high‐impact international journals [4, 5], hosting iMeta conferences and online forums [6, 7], and boosting academic sharing via social media [20]. Looking ahead, iMeta will continue to redefine the boundaries of biology and medicine through deep integration of informatics and metaomics, not merely as a publishing platform, but as a hub for interdisciplinary innovation and global collaboration.

## AUTHOR CONTRIBUTIONS

**Gousi Li**: Conceptualization; data curation; investigation; visualization; writing—original draft; funding acquisition; writing—review and editing. **Qiao Yu**: Methodology; Data curation; investigation; visualization; writing—original draft; writing—review and editing. **Zhihao Zhu, Xiaofang Yao, Yao Wang, Hao Luo, Defeng Bai, Yunyun Gao, Jiani Xun, Meiyin Zeng, Tianyuan Zhang, Tengfei Ma, Zi‐Ang Shen, Wei Han, Jiqiu Wu, Li‐Jing Bai**: Writing—original draft; project administration; writing—review and editing; investigation. **Kui Li, Suyin Feng, Ziheng Wang, Peng Luo, Ren‐You Gan, Gang Chen, Yulong Yin, Tong Chen, Yong‐Xin Liu:** Conceptualization, supervision, funding acquisition, project administration, writing—review and editing. All other authors: Writing—review and editing.

## CONFLICT OF INTEREST STATEMENT

Shuang‐Jiang Liu is the Editor‐in‐Chief of *iMeta*; Yong‐Xin Liu and Tong Chen are Executive Editors of *iMeta*. The other authors declare no conflicts of interest.

## ETHICS STATEMENT

No animals or humans were involved in this study.

## AI STATEMENT

ChatGLM (v5.3) was used for language polishing. All content has been verified for accuracy by the authors.

## Supporting information


**Figure S1**: Literature retrieval and analysis workflow.

**Table S1:** Included Web of Science Categories for biomedical literature collection (Set B).
**Table S2:** Annual publication counts of biomedical literature collection.
**Table S3:** Search‐term blocks and annual publication counts for the informatics literature collection within Set B.
**Table S4:** Search‐term blocks and annual publication counts for the omics literature collection within Set B.
**Table S5:** Bibliographic metadata and annual ranks of the 1000 annual high‐impact publications.
**Table S6:** All high‐frequency terms from the 1000 annual high‐impact publications (≥10 records).
**Table S7:** The retained terms used for co‐occurrence network analysis.

## Data Availability

The data and scripts used are saved in GitHub: https://github.com/yuqiao617/iMeta-Omics-Hotspot. The data of iMeta Conference 2026 are available on the meeting website: http://www.imeta.science/meeting/2026. Supplementary materials (methods, figures, tables, graphical abstract, slides, videos, Chinese translated version, and updated materials) may be found in the online DOI or iMeta Science http://www.imeta.science/.
