# Supplementary material for: iMeta: Biology and medicine in informatics and MetaOmics era
Source: Imeta. 2026 Nov 13:e70189. Online ahead of print. doi: 10.1002/imt2.70189 (PMC13760654; doi:10.1002/imt2.70189)
Supplement: Supplementary file 1 [file IMT2-9999-e70189-s001.docx]

Supporting Information to

**iMeta: Biology and Medicine in Informatics and MetaOmics Era**

**Running title**: **iMeta: BioMed Informatics and MetaOmics**

Gousi Li^1#^, Qiao Yu^1#^, Zhihao Zhu^1#^, Xiaofang Yao^1#^, Yao Wang^1#^, Hao Luo^1#^, Defeng Bai^1#^, Yunyun Gao^2#^, Jiani Xun^1#^, Meiyin Zeng^1#^, Tianyuan Zhang^3#^, Tengfei Ma^4#^, Zi-Ang Shen^1#^, Wei Han^5#^, Jiqiu Wu^6#^, Li-Jing Bai^7#^, Feiying Zhu^8^, Chun‐Sheng Hou^9^, Lin Zhang^10^, Cheng Gao^11^, Renhong Yan^12^, Yi Song^13^, Guirong Wang^7^, Shan‐shan Xu^14^, Weihua Pan^1^, Hao Zheng^15^, Yue Sheng^16^, Qingsong Ye^17^, Jia‐Hui Liu^7^, Chun Yin^18^, Jialong Cao^19^, Dong‐Rui Chen^20^, Hanqing Chen^21^, Hu Chen^3^, Ji‐Kang Chen^9^, Jiani Chen^22^, Mingliang Chen^23^, Ruibing Chen^24^, Lei Cheng^25^, Die Dai^26^, Xi‐Jian Dai^27^, Jianjun Deng^28^, Xile Deng^29^, Na Ding^30^, XiaDing^31^, Zequan Ding^32^, Xuan Dong^33^, Yong‐Zhen Dong^34^, Jiajia Duan^35^, Yan Gao^36^, Yi‐Zhou Gao^37^, Ya‐Ting Ge^38^, Wenping Gong^39^, HarvestF. Gu^40^, Rui Guo^41^, Xiang Guo^42^, Ying‐ying Han^43^, Jiu‐Xing He^44^, Xiaoqing He^45^, Haiming Hu^46^, Jianhua Huang^22^, Roujie Huang^47^, Yuansheng Huang^3^, Di Jiang^48^, Mengxi Jiang^49^, Tao Jiang^50^, Juan Lai^51^, Bo Li^52^, Huibo Li^53^, Jian‐Feng Li^54^, Jian Li^55^, Ruichao Li^56^, Shuai Li^57^, Wei Li^58^, Xiaojing Li^3^, Xuemeng Li^59^, Yin Li^60^, Zhan‐zhan Li^61^, Jie Liao^62^, Xing‐Hua Liao^63^, Biao Liu^64^, Bingqi Liu^65^, Sheng Liu^66^, Tianyang Liu^67^, Wen‐Hui Liu^16^, Yi Liu^68^, Zhipeng Liu^69^, Hong‐Ye Lu^70^, Hui Lu^71^, Xiao Luo^72^, Haiquan Lu^73^, Wei Ma^74^, Hongfeng Mao^75^, Wenjun Mao^76^, Ming Nie^77^, Huili Pang^78^, Yan Pang^75^, Ziqin Pang^44^, Hui Qu^79^, Cheng Shen^58^, Chuankui Song^80^, Xiaoming Song^81^, Qi Su^82^, Hao Sun^83^, Xinyi Sun^84^, Yan Sun^85^, Xiaoqiang Tang^86^, Bowen Wang^87^, Dingjie Wang^88^, Dong Wang^89^, Gui‐Wei Wang^90^, Hanfei Wang^91^, Nanqi Wang^92^, Shixiang Wang^93^, Tong Wang^94^, Xi Wang^95^, Xinyuan Wang^75^, Yufeng Wang^96^, Yongjun Wei^97^, Shu Wen^98^, Peng Wu^99^, Shengbo Wu^100^, Yuan Xiang^101^, Haiqing Xiao^102^, Jian Xiao^103^, Weihua Xiao^104^, Jun Yan^105^, Bingsheng Yang^106^, Haixia Yang^107^, Xin Yang^108^, Ze‐Wei Yang^51^, Shi‐Jie Yao^109^, Hengchuang Yin^110^, Yangyan Yin^111^, Li Yu^112^, Xinxin Yu^94^, Can Zhang^58^, Chi Zhang^113^, Deyong Zhang^114^, Jian Zhang^115^, Jinping Zhang^116^, Jingsong Zhang^117^, Peng Zhang^118^, Yu Zhang^119^, Pingsen Zhao^120^, Tianyi Zhao^52^, Xue‐Ming Zhao^121^, Yuchao Zhao^122^, Yucheng Zhao^123^, Ruihao Zhou^124^, Tao Zhou^125^, Xin Zhou^11^, Yongmei Zhou^44^, Siyuan Zhu^9^, Xiusheng Zhu^126^, Yilin Xu^58^, Xiong‐Feng Ma^9^, Lianyang Bai^127^, Zhenmin Bao^128^, Shuang‐Jiang Liu^129^, KuiLi^7*^, Suyin Feng^130*^, Ziheng Wang^131*^, Peng Luo^132*^, Ren‐You Gan^133*^, Gang Chen^134*^, Yulong Yin^135*^, Tong Chen^136*^, Yong‐Xin Liu^1*^

^1^State Key Laboratory of Tropical Crop Breeding, Genome Analysis Laboratory of the Ministry of Agriculture and Rural Affairs, Agricultural Genomics Institute at Shenzhen, Chinese Academy of Agricultural Sciences, Shenzhen, Guangdong, China

^2^School of Ecology and Nature Conservation, Beijing Forestry University, Haidian District, Beijing, China

^3^Benagen Institute, Wuhan, Hubei, China

^4^State Key Laboratory of Herbage Improvement and Grassland Agro-ecosystems, Centre for Grassland Microbiome, College of Pastoral Agriculture Science and Technology, Lanzhou University, Lanzhou, Gansu, China

^5^College of Food Science and Engineering, Central South University of Forestry and Technology, Changsha, Hunan, China

^6^CAS Key Laboratory of Pathogen Microbiology and Immunology, Institute of Microbiology, Chinese Academy of Sciences (CAS), Beijing, China

^7^Agricultural Genomics Institute at Shenzhen, Chinese Academy of Agricultural Sciences, Shenzhen, Guangdong, China

^8^Hunan Academy of Agricultural Sciences, Changsha, China

^9^Institute of Bast Fiber Crops, Chinese Academy of Agricultural Sciences, Changsha, Hunan, China

^10^Hubei Shizhen Laboratory, School of Basic Medical Sciences, Hubei University of Chinese Medicine, Wuhan, China

^11^State Key Laboratory of Microbial Diversity and Innovative Utilization, Institute of Microbiology, Chinese Academy of Sciences, Beijing, China

^12^Southern University of Science and Technology, Shenzhen, Guangdong, China

^13^Shenzhen Key Laboratory of Plant Genetic Engineering and Molecular Design, Institute of Plant and Food Science, Department of Biology, School of Life Sciences, Southern University of Science and Technology, Shenzhen, China

^14^Institute of Agro-Products Processing Research Anhui Academy of Agricultural Sciences, Hefei, China

^15^College of Food Science and Nutritional Engineering, China Agricultural University, Beijing, China

^16^ The Second Xiangya Hospital of Central South University, Changsha, Hunan, China

^17^Center of Regenerative Medicine, Department of Stomatology, Renmin Hospital of Wuhan University, Wuhan, China

^18^State Key Laboratory of Genetics and Development of Complex Phenotypes, Institute of Plant Biology, School of Life Sciences, Fudan University, Shanghai, China

^19^Equus Research Center, Inner Mongolia Agricultural University, Hohhot, China

^20^State Key Laboratory for Animal Disease Control and Prevention, South China Agricultural University, Guangzhou, Guangdong, China

^21^Beijing Key Laboratory of Environment and Aging, Department of Nutrition and Food Hygiene, School of Public Health, Capital Medical University, Beijing, China

^22^Zhejiang Key Laboratory of Biology and Ecological Regulation of Crop Pathogens and Insects, Institute of Insect Sciences, College of Agriculture and Biotechnology, Zhejiang University, Hangzhou, Zhejiang, China

^23^Department of Clinical Nutrition, Southwest Hospital, Army Medical University (Third Military Medical University), Chongqing, China

^24^Department of Pharmaceutical Botany, School of Pharmacy, Naval Medical University, Shanghai, China

^25^State Key Laboratory for Vegetation Structure, Function and Construction, MOE Key Laboratory of Biosystems Homeostasis & Protection, College of Life Sciences, Zhejiang University, Hangzhou, China

^26^Chinese Medicine Guangdong Laboratory, Guangzhou University of Chinese Medicine, Hengqin, Guangdong, China

^27^Department of Medical Imaging, The First Affiliated Hospital of Gannan Medical University, Ganzhou City, Jiangxi Province, China

^28^State Key Laboratory of Vegetable Biobreeding, Institute of Vegetables and Flowers, Chinese Academy of Agricultural Sciences, Beijing, China

^29^Yuelushan Laboratory, Hunan Academy of Agricultural Sciences, Changsha, China

^30^Harbin Medical University, Harbin, China

^31^Nanchang University, Nanchang, China

^32^Department of Pediatric Surgery, Children's Hospital of Nanjing Medical University, Nanjing, Jiangsu Province, China

^33^Yellow Sea Fisheries Research Institute, Chinese Academy of Fishery Sciences, Qingdao, China

^34^Academy of Food Interdisciplinary Science, Dalian Polytechnic University, Dalian, China

^35^Luoyang Key Laboratory of Zebrafish Toxicology Research, Luoyang Key Laboratory of Transplantation and Immunological Studies for Hematological Diseases, Department of Clinical Laboratory, the First Affiliated Hospital, College of Clinical Medicine of Henan University of Science and Technology, Luoyang, Henan, China

^36^Harbin Institute of Technology, Harbin, China

^37^Department of Environmental Hygiene, S chool of Public Health, Harbin Medical University, Harbin, China

^38^State Key Laboratory of Bioactive Substance and Function of Natural Medicines, Institute of Materia Medica, Chinese Academy of Medical Sciences and Peking Union Medical College, Beijing, China

^39^Senior Department of Tuberculosis, Chinese PLA General Hospital, Beijing, China

^40^Laboratory of Molecular Medicine, School of Basic Medicine and Clinical Pharmacy, China Pharmaceutical University, Nanjing, Jiangsu, China

^41^State Key Laboratory for Quality and Safety of Agro-Products, Institute of Environment, Resource, Soil and Fertilizer, Zhejiang Academy of Agricultural Sciences, Hangzhou, China

^42^Department of Pathogen Biology, School of Basic Medical Sciences, Henan University, Kaifeng, China

^43^College of Food Science and Engineering, Shandong Agricultural University, Tai'an, Shandong, China

^44^Xianghu Laboratory, Hangzhou, China

^45^State Key Laboratory of Efficient Production of Forest Resources, College of Biological Sciences and Technology, Beijing Forestry University, Beijing, China

^46^School of Laboratory Medicine, Hubei University of Chinese Medicine, Wuhan, Hubei, China

^47^National Clinical Research Center for Women's Health and Obstetric and Gynecologic Diseases, Department of Obstetrics and Gynecology, State Key Laboratory of Common Mechanism Research of Major Diseases, Peking Union Medical College Hospital, Chinese Academy of Medical Sciences and Peking Union Medical College, Beijing, China

^48^Henan University of Technology, Zhengzhou, Henan, China

^49^Department of Pharmacology, School of Basic Medical Sciences, Capital Medical University, Fengtai District, Beijing, China

^50^Faculty of Computing, Harbin Institute of Technology, Harbin, China

^51^GeneMind Biosciences Company Limited, Shenzhen, Guangdong, China

^52^School of Medicine and Health, Harbin Institute of Technology, Harbin, Heilongjiang, China

^53^Department of Pharmacy, Peking University Third Hospital, Beijing, China

^54^Shanghai Institute of Hematology, State Key Laboratory of Medical Genomics, National Research Center for Translational Medicine at Shanghai, Ruijin Hospital, Shanghai Jiao Tong University School of Medicine, Shanghai, China

^55^Jiangxi Province Key Laboratory of Pharmacology of Traditional Chinese Medicine, State Key Laboratory of New Targets Discovery and Drug Development for Major Diseases, School of Pharmacy, Gannan Medical University, Ganzhou, Jiangxi, China

^56^Jiangsu Co-Innovation Center for Prevention and Control of Important Animal Infectious Diseases and Zoonoses, College of Veterinary Medicine, Yangzhou University, Yangzhou, China

^57^Laboratory of Brewing Microbiology and Applied Enzymology, Key Laboratory of Industrial Biotechnology of Ministry of Education, School of Biotechnology, Jiangnan University, China

^58^Shenzhen Bay Laboratory, Pingshan Translational Medicine Center, Shenzhen, China

^59^Zhanjiang Key Laboratory of Human Microecology and Clinical Translation Research, the Marine Biomedical Research Institute, School of Basic Medicine, Guangdong Medical University, Zhanjiang, Guangdong, China

^60^ The International Science and Technology Cooperation Base (Genetic Engineering) of Chinese Ministry of Science and Technology, the Key Laboratory of Molecular Biophysics of Chinese Ministry of Education, College of Life Science and Technology, Huazhong University of Science and Technology, Wuhan, China

^61^Academy of Chinese Medicine Sciences, Henan University of Chinese Medicine, China

^62^State Key Laboratory of Chinese Medicine Modernization, Innovation Center of Yangtze River Delta, Zhejiang University, Jiaxing, Zhejiang, China

^63^Institute of Biology and Medicine, College of Life and Health Science, Wuhan University of Science and Technology, Wuhan, China

^64^Hunan Institute of Microbiology, Hunan Academy of Agricultural Sciences, Changsha, Hunan, China

^65^Jiangxi Provincial Key Laboratory of Pest and Disease Control of Featured Horticultural Plants, Gannan Normal University, Ganzhou, Jiangxi, China

^66^ The Seventh Affiliated Hospital of Sun Yat-sen University, Shenzhen, Guangdong, China

^67^Department of Microbiology, College of Life Science, Shandong Agricultural University, Tai'an, Shandong, China

^68^Collaborative Innovation Center for Zhejiang Marine High-efficiency and Healthy Aquaculture, School of Marine Sciences, Ningbo University, Ningbo, Zhejiang, China

^69^State Key Laboratory of Herbage Improvement and Grassland Agro-Ecosystems, College of Pastoral Agriculture Science and Technology, Lanzhou University, Lanzhou, Gansu, China

^70^Stomatology Hospital, School of Stomatology, Zhejiang University School of Medicine; Clinical Research Center for Oral Diseases of Zhejiang Province; Key Laboratory of Oral Biomedical Research of Zhejiang Province; Cancer Center of Zhejiang University, Hangzhou, Zhejiang, China

^71^Department of Pharmacy, Shanghai Tenth People's Hospital, School of Medicine, Tongji University, Shanghai, China

^72^Hunan Research Center of the Basic Discipline for Cell Signaling, College of Biology, Hunan University, Changsha, Hunan, China

^73^Advanced Medical Research Institute, Cheeloo College of Medicine, Shandong University, Jinan, Shandong, China

^74^State Key Laboratory of North China Crop Improvement and Regulation, Key Laboratory of Vegetable Germplasm Innovation and Utilization of Hebei, College of Horticulture, Hebei Agricultural University, Baoding, Hebei, China

^75^State Key Laboratory of Metabolic Dysregulation & Prevention and Treatment of Esophageal Cancer, Shanghai Key Laboratory of Diabetes Mellitus, Department of Endocrinology and Metabolism, Shanghai Diabetes Institute, Shanghai Clinical Center for Diabetes, Shanghai Sixth People's Hospital Affiliated to Shanghai Jiao Tong University School of Medicine, Shanghai, China

^76^Department of Thoracic Surgery, the Affiliated Wuxi People's Hospital of Nanjing Medical University, Wuxi People's Hospital, Wuxi Medical Center, Nanjing Medical University, Wuxi, China

^77^National Observations and Research Station for Wetland Ecosystems of the Yangtze Estuary, School of Life Sciences, Fudan University, Shanghai, China

^78^State Key Laboratory of Cotton Bio-breeding and Integrated Utilization, School of Agriculture and Biomanufacturing, Zhengzhou University, Zhengzhou, China

^79^Institute of Grassland Research, Chinese Academy of Agricultural Sciences, Hohhot, Inner Mongolia, China

^80^State Key Laboratory of Tea Plant Germplasm Innovation and Resource Utilization, Anhui Agricultural University, Hefei, China

^81^School of Life Sciences, North China University of Science and Technology, Tangshan, Hebei, China

^82^Department of Medicine and Therapeutics, Chinese University of Hong Kong, China

^83^Wolfson Institute for Biomedical Research, UCL Division of Medicine, University College London, Gower St, London, UK

^84^Mingqian Institute of Microbiology, Suzhou, Jiangsu, China

^85^Chinese National Microbiology Data Center (NMDC), Beijing, China

^86^Key Laboratory of Birth Defects and Related Diseases of Women and Children of MOE, West China Second University Hospital, Sichuan University, Chengdu, China

^87^Guojiu Research Institute, School of Food and Health, Beijing Technology and Business University, Beijing, China

^88^Institute for Math & AI, Wuhan University, Wuhan, China

^89^College of Veterinary Medicine, Shandong Agricultural University, Tai'an, Shandong, China

^90^National Engineering Research Center for Efficient Utilization of Soil and Fertilizer Resources, College of Resources and Environment, Shandong Agricultural University, Taian, Shandong, China

^91^College of Animal Science and Technology, Yangtze University, Jingzhou, Hubei, China

^92^National Citrus Engineering Research Center, Chongqing Key Laboratory of Citrus, Citrus Research Institute, Southwest University, Chongqing, China

^93^Department of Biomedical Informatics, School of Life Sciences, Central South University, Changsha, Hunan, China

^94^Hunan Kaiyou Biotechnology Co., Ltd., Changsha, Hunan, China

^95^State Key Laboratory of Reproductive Medicine and Offspring Health, Nanjing Medical University, Nanjing, China

^96^College of Animal Science, Inner Mongolia Agricultural University, Hohhot, Inner Mongolia, China

^97^School of Pharmaceutical Sciences, Key Laboratory of Advanced Drug Preparation Technologies, Ministry of Education, Zhengzhou University, Zhengzhou, China

^98^Wensyn Life Science Co., Ltd., Wuxi, China

^99^Department of Nephrology, The First Affiliated Hospital of Zhengzhou University, Zhengzhou, Henan, China

^100^Zhejiang Institute of Tianjin University (Shaoxing), Shaoxing, China

^101^Department of Medical Laboratory, The Central Hospital of Wuhan, Tongji Medical College, Huazhong University of Science and Technology, Wuhan, Hubei, China

^102^State Key Laboratory of Vaccines for Infectious Diseases, National Institute of Diagnostics and Vaccine Development in Infectious Diseases, Xiang An Biomedicine Laboratory, School of Life Sciences & School of Public Health, Xiamen University, Xiamen, Fujian, China

^103^College of Agriculture, Hunan Agricultural University, Changsha, China

^104^Beilun People's Hospital, Ningbo, Zhejiang, China

^105^College of Agronomy and Biotechnology, China Agricultural University, Beijing, China

^106^Department of Joint and Orthopedics, Zhujiang Hospital, Southern Medical University, Guangzhou, Guangdong, China

^107^College of Food Science and Nutritional Engineering, China Agricultural University, China

^108^State Key Laboratory for Quality and Safety of Agro-Products, Ningbo University, Ningbo, China

^109^School of Urban Construction & Safety Engineering, Shanghai Institute of Technology, Shanghai, China

^110^Xinjiang Technical Institute of Physics and Chemistry, Chinese Academy of Sciences, Urumqi, Xinjiang, China

^111^College of Animal Science and Technology, Guangxi University, Nanning, Guangxi, China

^112^School of Life Sciences, State Key Laboratory for Conservation and Utilization of Bio-Resource in Yunnan, Yunnan University, Kunming, China

^113^Department of Orthopaedics, The Second Qilu Hospital of Shandong University, Shandong University Centre for Orthopaedics, Cheeloo College of Medicine, Shandong University, Jinan, China

^114^State Key Laboratory of Hybrid Rice, Institute of Plant Protection, Hunan Academy of Agricultural Sciences, Changsha, China

^115^Department of Gastroenterology, the First Affiliated Hospital (Southwest Hospital) of Third Military Medical University (Army Medical University), Chongqing, China

^116^Pediatric Department, Sixth People's Hospital, Jiao Tong University, Shanghai, China

^117^Faculty of Military Health Services, Naval Medical University, Shanghai, China

^118^Department of Environment, College of Environment and Resources, Xiangtan University, Xiangtan, China

^119^Guangdong Provincial Key Laboratory of Marine Biotechnology, Department of Biology, College of Science, Shantou University, Shantou, Guangdong, China

^120^Yuebei People's Hospital Affiliated to Shantou University Medical College, Shaoguan, Guangdong, China

^121^Institute of Animal Science, Chinese Academy of Agricultural Sciences, Beijing, China

^122^College of Animal Science and Technology, Beijing University of Agriculture, Beijing, China

^123^School of Pharmacy, Nanjing University of Chinese Medicine, Nanjing, China

^124^Department of Anesthesiology, The Research Units of West China (2018RU012)-the Anesthesiology & Intelligence Research Collaboration Group (AIR-CG), West China Hospital, Sichuan University, Chengdu, Sichuan, China

^125^Faculty of Life and Health Sciences, Shenzhen University of Advanced Technology, Shenzhen, China

^126^Department of Genetics and Bioinformatics, School of Life Sciences, Anhui Medical University, Hefei, Anhui, China

^127^Hunan Academy of Agricultural Sciences, Changsha, Hunan, China

^128^MOE Key Laboratory of Marine Genetics and Breeding, Qingdao Institute of Blue Seed Industry, Ocean University of China, Qingdao, China

^129^State Key Laboratory of Microbial Technology, Shandong University, Jinan, Shandong, China

^130^Cardio-Cerebral Vascular Disease Prevention and Treatment Innovation Center, Donghai County People's Hospital, (Affiliated Kangda College of Nanjing Medical University), Lianyungang, Jiangsu, China

^131^Department of Rehabilitation Sciences, Faculty of Health and Social Sciences, The Hong Kong Polytechnic University, Hung Hom, Hong Kong SAR, China

^132^Department of Oncology, Zhujiang Hospital, Southern Medical University, Guangzhou, Guangdong, China

^133^Department of Food Science and Nutrition, The Hong Kong Polytechnic University, Kowloon, Hong Kong SAR, China

^134^Hubei University of Chinese Medicine, Hubei Shizhen Laboratory, Department of Geriatrics, Affiliated Hospital of Hubei University of Chinese Medicine, Wuhan, China

^135^Hunan Provincial Key Laboratory of Animal Nutritional Physiology and Metabolic Process, National Engineering Laboratory for Pollution Control and Waste Utilization in Livestock and Poultry Production, Institute of Subtropical Agriculture, Chinese Academy of Sciences, Changsha, Hunan, China

^136^State Key Laboratory for Quality Ensurance and Sustainable Use of Dao-di Herbs, National Resource Center for Chinese Materia Medica, Institute of Chinese Materia Medica, China Academy of Chinese Medical Sciences, Beijing, China

^#^These authors contributed equally: Gousi Li, Qiao Yu, Zhihao Zhu, Xiaofang Yao, Yao Wang, Hao Luo, Defeng Bai, Yunyun Gao, Jiani Xun, Meiyin Zeng, Tianyuan Zhang, Tengfei Ma, Zi-Ang Shen, Wei Han, Jiqiu Wu, Li-Jing Bai

^*^Correspondence: [likui@caas.cn](mailto:likui@caas.cn) (Kui Li), [fengsuyin@njmu.edu.cn](mailto:fengsuyin@njmu.edu.cn) (Suyin Feng), [zi-heng.wang@connect.polyu.hk](mailto:zi-heng.wang@connect.polyu.hk) (Ziheng Wang), [luopeng@smu.edu.cn](mailto:luopeng@smu.edu.cn) (Peng Luo), [renyou.gan@polyu.edu.hk](mailto:renyou.gan@polyu.edu.hk) (Ren-You Gan), [chengang@hbucm.edu.cn](mailto:chengang@hbucm.edu.cn) (Gang Chen), [yinyulong@isa.ac.cn](mailto:yinyulong@isa.ac.cn) (Yulong Yin), [chent@nrc.ac.cn](mailto:chent@nrc.ac.cn) (Tong Chen), [liuyongxin@caas.cn](mailto:liuyongxin@caas.cn) (Yong-Xin Liu)

**METHODS**

**Bibliometric search strategy**

Publications were retrieved from the Web of Science (WoS) Core Collection (SCI-EXPANDED). The initial search was performed on 8 August 2026, the final search sets were reconstructed on 9 August 2026, and the selected records for hotspot analysis were exported on 10 August 2026. Articles or reviews in English with Final Publication Year from 2016 to 2025 were included.

For the biomedical literature collection (Set B), a manually reviewed list of 86 WoS Categories covered fields in biology and medicine was used for bibliometric search (Table S1). Set B contains 8,919,492 records (Table S2).

For the informatics literature collection (Set I) and the omics literature collection (Set O), the corresponding search terms (Table S3 and S4) were applied separately to titles (TI), abstracts (AB), and author keywords (AK), using TI = (term block) OR AB = (term block) OR AK = (term block). Set I and Set O were intersected with Set B. The resulting B ∩ (I ∪ O) with 1,312,335 records was used to calculate the adoption rate of informatics and metaomics in biology and medicine.

Annual publication counts and country/region records were retained for the search sets.

**Data processing and bibliometric indicators**

Publications in Set I were grouped into six non-exclusive categories, including AI, bioinformatics, computational drug discovery, clinical and imaging informatics, digital health, and big data; publications in Set O were grouped into nine non-exclusive categories, including genomics, transcriptomics, proteomics, metabolomics, metagenomics/microbiome, single-cell omics, spatial omics, multi-omics, and other omics, according to the TI, AB, and AK term blocks in Tables S3 and S4. The other omics contributed to Set O was included in Figure 1A, E, and F but excluded in Figure B‒D. Annual adoption of the categories indicates the proportion of publications in each category relative to the total number of publications in Set B. Compound annual growth rates (CAGRs) of the categories were calculated as [(N_2025_/N_2016_)^(1/9)−1]×100%, where N_2016_ and N_2025_ are the numbers of records assigned to the corresponding category in 2016 and 2025. The participation proportion of each country/region was calculated as the number of participation records from that country/region divided by the total number of participation records across all countries/regions. Pairwise collaboration was defined by co-occurrence of two countries/regions within the same record, the frequencies of which were calculated for the top 15 countries/regions, and the union of the top 30 pairs in the five-year (2021‒2025) and ten-year (2016‒2025) windows was visualized.

To obtain 1000 annual high-impact informatics- and omics-enabled studies for hotspot analysis, annual quotas were assigned in proportion to the size of B ∩ (I ∪ O) using the largest-remainder method. The quotas for 2016–2025 were 49, 53, 60, 73, 90, 113, 127, 123, 139, and 173, respectively. Within each year, records were ranked in descending order of cumulative WoS citations measured from publication until the date of export, and the top-ranked records up to the yearly quota were retained (Table S5). Terms in the title and abstract were analysed in VOSviewer 1.6.21 using binary counting [1]. Of 25,928 initially identified terms, 590 occurred in at least 10 records (Table S6). These terms were manually reviewed to remove uninformative terms and merge spelling variants, abbreviations, and synonyms into standardized forms. The final VOSviewer network contains 153 terms in five clusters (Table S7). Although studies were ranked within each publication year to avoid cross-year citation bias, within-year differences due to publication month may underestimate the impact of the most recent publications, and future updates with more comparable citation windows may capture them more fully.

For paper-level theme analyses, a period was added after titles lacking terminal punctuation to preserve the title–abstract sentence boundary. Terms were re-extracted in VOSviewer 1.6.21 with the final thesaurus, binary counting and the same occurrence threshold. Term–document membership was recovered using indicators that assigned 1 to one record and 0 to all others. A positive mean indicator score identified a term in that record. The analysis retained the confirmed 153 terms, original clusters and coordinates. All term frequencies and 11,628 pairwise co-occurrence counts, including zeros, matched the final network. Records containing at least one retained term from a cluster were assigned to that theme. This yielded 968 classified records and 1,968 non-exclusive theme–paper links; 663 records belonged to multiple themes and 32 remained unclassified. Figure 2B shows shared-record counts and Jaccard similarity (intersection divided by union). In Figure 2C, each record assigned to k themes contributes 1/k to each, so annual totals equal the classified sample size. Figure 2D reports theme coverage using the full annual sample as denominator, peak years and the largest year-on-year increases in percentage points. Figure 2E uses the same denominator for annual occurrence percentages of the ten most frequent terms per cluster. Cluster 5 has nine terms and is omitted from Figure 2E. These descriptive results only characterize the citation-ranked publications.

**Data visualization**

The term co-occurrence network and its clustering were generated via VOSviewer 1.6.21. Node positions and clusters were retained during graphical editing; dashed ellipses indicate visual cluster cores but not confidence regions. Other panels were rendered using R or Python scripts saved in GitHub https://github.com/yuqiao617/iMeta-Omics-Hotspot.

**REFERENCES**

1. van Eck NJ, Waltman L. Software survey: VOSviewer, a computer program for bibliometric mapping. Scientometrics. 2010;84:523–538. https://doi.org/10.1007/s11192-009-0146-3.

**SUPPLEMENTARY FIGURE**

Figure S1. Literature retrieval and analysis workflow. The biomedical literature collection (Set B), its intersections with informatics (I) and omics (O), and the corresponding subsets supported the statistical analyses in Figure 1. The combined candidate set B ∩ (I ∪ O), containing 1,312,335 records, provided the sampling frame for Figure 2. Annual proportional allocation and within-year ranking by cumulative citations yielded 1,000 unique records. Title-and-abstract term extraction, thesaurus normalization, semantic review, and VOSviewer clustering produced 153 terms in five clusters (Figure 2A). Document membership was recovered for all 153 retained terms after preserving the sentence boundary between title and abstract, assigning 968 papers to at least one theme; 32 papers remained unclassified. These assignments supported the downstream analyses in Figure 2B–E. Cluster 5 was retained in Figure 2A–D and omitted from Figure 2E.
